# Ocular manifestation of vertical transmission of dengue: case report

**DOI:** 10.5935/0004-2749.2022-0107

**Published:** 2022-10-19

**Authors:** Rubens Camargo Siqueira, Igor Neves Coelho, João Pedro Romero Braga, Moises Moura de Lucena, Victor C. F. Bellanda, Anita Agarwal, Rodrigo Jorge

**Affiliations:** 1 Division of Ophthalmology, Faculdade de Medicina de Ribeirão Preto, Universidade de São Paulo, Ribeirão Preto, SP, Brazil; 2 Department of Ophthalmology, Sir Charles Gairdner Hospital, Western Australia, Australia; 3 West Coast Retina, San Francisco, CA, USA

**Keywords:** Dengue, Pregnancy complication, infectious, Infectious disease transmission, vertical, Eye manifestations, Retinal hemorrhage, Vitreous hemorrhage, Human, Infant, newborn, diseases, Case reports, Dengue, Complicações infecciosas na gravidez, Transmissão vertical de doenças infecciosas, Manifestações oculares, Hemorragia retiniana, Hemorragia vítrea, Humanos, Doenças do recém-nascido, Relatos de casos

## Abstract

A 7-week-old male delivered by cesarean section presented with a positive serology for dengue along with preretinal and retinal hemorrhages, vitreous opacities and cotton wool spots. The patient and his mother had positive serologies for Non Structural Protein 1 (NS1) by ELISA. Retinal and vitreous findings improved over a sixteen-week period. Spectral domain optical coherence tomography (OCT) showed preserved macular architecture. In this case report, we suggest that retinal and vitreous changes may be the ocular presenting features of vertically transmitted dengue in newborns, and that those findings may resolve with no major structural sequelae.

## Abstract (pt)

Neonato de 7 semanas, do sexo masculino, nascido de parto cesárea, apresentou sorologia positiva para dengue com hemorragias retinianas e pré-retinianas, opacidades vítreas e manchas algodonosas. O paciente e sua mãe haviam apresentado sorologias positivas para *Non Structural Protein 1* através de ELISA. Achados na retina e no vítreo melhoraram em um período de dezesseis semanas. O exame de tomografia de coerência óptica de domínio espectral demonstrou arquitetura macular preservada. Neste relato de caso, sugerimos que alterações na retina e no vítreo podem ser os achados oculares aparentes em neonatos com infecção vertical por dengue, e que estes podem se resolver sem maiores sequelas estruturais.

## INTRODUCTION

Dengue is considered to be one of the most common tropical arbovirus diseases, with approximately 100,000 persons infected per year worldwide. It is a mostly self-limited systemic viral infection transmitted between humans via insect bites^(1)^.

Dengue infection during pregnancy is concerning for a higher risk of maternal mortality, in addition to increasing the risk of cesarean delivery and postpartum bleeding. Approximately 90% of pregnancies recorded annually occur in endemic and epidemic areas of arbovirus diseases, and approximately 2.8% of Brazilian pregnant women were positive by serology for dengue during the 2008-2009 outbreak^(2)^. Other complications include perinatal death, spontaneous abortion, premature delivery and low birthweight^(2)^. Published reports of congenital infections have emphasized the fact that dengue virus infection compromises newborns whose mothers become infected towards the end of pregnancy. Some studies have described isolating the virus from blood or in bone marrow samples^(2)^. There are few reports of vertical transmission of dengue, and consequently little information about the behavior of the disease with this type of transmission is available^(3)^.

Several reports have demonstrated ophthalmological manifestations of dengue acquired during adulthood, including retinal hemorrhages, cotton wool spots, white centered hemorrhages and optic disc swelling^(4,5,6,7)^. We report herein a 6-week old baby with retinal findings similar to dengue retinopathy; to the best of our knowledge, this is the first described case of ocular manifestation consequent to vertical transmission from mother to child.

## CASE REPORT

A previously healthy woman in her 38th week of pregnancy exhibited signs and symptoms of dengue with fever, arthralgia and myalgia. The clinical diagnosis of dengue was confirmed by serology. On the day of diagnosis she had a platelet count of 180,000 cells/mm^3^. Over the next five days, with clinical and hematological worsening, there was a significant and abrupt reduction of the platelet count to 3,000 cells/mm^3^. With progression to severe disease, the patient needed hospitalization. At 38 weeks and 6 days of pregnancy she had premature rupture of membranes with consequent fetal distress, requiring an emergency cesarean delivery.

**Figure 1:**
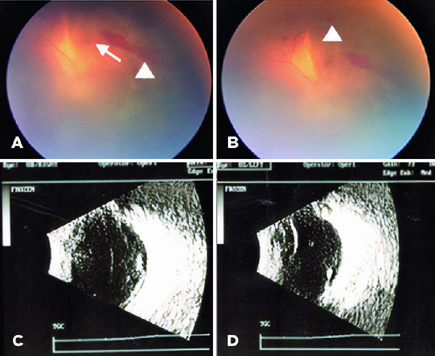
At presentation, color fundus pictures revealed vitreous opacities (gray vitreous hemorrhage) and retinal (arrow) and preretinal hemorrhages (arrowheads) (A, B). Ultrasound showed vitreous opacities corresponding to vitreous hemorrhage. (C, D).

The baby boy was born with a platelet count of 13,000/mm^3^. He suffered two episodes of cardiorespiratory arrest and remained in the ICU for 29 days with a diagnosis of fetal anoxia. At 7 weeks of age, with a positive serology for dengue, he was referred to the retina and vitreous service for further investigation and management. On admission, fundus examination of the right eye (OD) revealed vitreous hemorrhage, and preretinal and retinal hemorrhages in all four quadrants. Examination of the left eye (OS) showed similar findings, with preretinal and retinal hemorrhages only in the superior equatorial retina. Ocular ultrasound revealed punctiform vitreous echoes of low echogenicity suggestive of an inflammatory/hemorrhagic process in both eyes (Figures 1 A-D).

**Figure 2:**
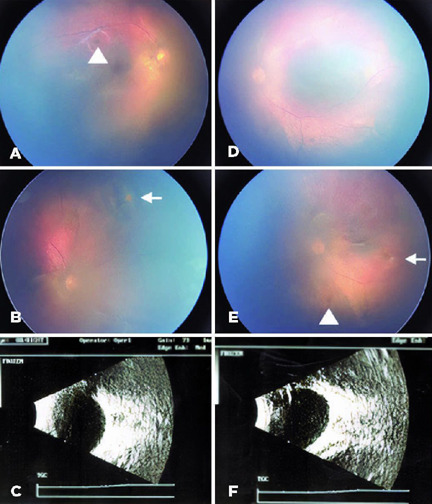
Color fundus pictures showing improvement of preretinal hemorrhages. White dots suggestive of infarction area are visible in both eyes (arrows); as well as red dots suggestive of residual bleeding (arrowheads). (A-D): Ultrasound showed reduction of vitreous opacities in both eyes. (E, F).

**Figure 3:**
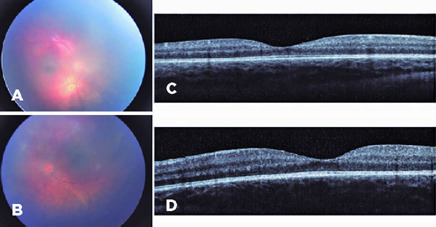
Sixteen weeks after presentation, fundus color pictures showed improvements of vitreous opacities and retinal hemorrhages (A, B). Optical coherence tomography showed preservation of the foveal architecture in both eyes. (C, D).

The retina team opted for an expectant management. Six weeks after presentation, there was a decrease of vitreous opacities and preretinal and retinal hemorrhages in both eyes. There was also one cotton wool spot in the superior nasal retinal of OD and one in the temporal equatorial retina of OS. The ultrasound confirmed reduction of vitreous opacities (Figures 2 A-F). Sixteen weeks after admission, there was further improvement of vitreous opacities and macular OCT revealed a preserved foveal contour, with grossly preserved architecture, and normal central subfield thickness (CSFT) for age (206 µm in OD and 211 µm in OS) (Figures 3 A-D).

## DISCUSSION

Based on a medline search, this is the first newborn ocular manifestation of dengue fever as a result of vertical transmission. Infection during pregnancy can affect fetal development, cause perinatal complications or transmit the virus to the fetus. Rigorous obstetrical monitoring is essential^(3)^. Paixão et al. conducted a systematic review of 16 studies of fetal complications in patients infected with dengue during pregnancy and selected 8 of them for meta-analysis, involving a total of 292 women exposed to the virus during pregnancy. The review revealed an association between the disease and abortion (OR 3.51), fetal death (RR 6.7), premature birth (OR 1.71), and low birth weight (OR 1.41)^(2)^. Our patient was of low birth weight, born from an emergency cesarean section due to fetal distress, with cardiac and respiratory complications, fetal anoxia, and significant reduction in platelet count.

In our report, the 7-week-old baby had retinal and preretinal hemorrhages, cotton wool spots suggestive of small vessel occlusions, and mild vitritis, findings previously described in adults with dengue fever retinopathy^(4,6)^. Other known ophthalmic findings are hypopyon uveitis, increased intraocular pressure, maculopathy, vasculitis, retinal hemorrhages, cotton wool spots, vascular occlusions, serous retinal detachment, and optic disc edema.

We propose the thrombocytopenia secondary to the dengue infection, and possibly dengue vasculitis, were likely responsible for the retinal and vitreous hemorrhages in this baby. There are several potential causes of retinal hemorrhages in neonates. Hemorrhages at birth may occur after traumatic deliveries^(8)^, or be associated with sepsis, shaken baby syndrome or intracranial hemorrhage. Our patient was born by cesarean section at 38 weeks of gestation with a birthweight of 3500g; and no serologic or clinical evidence of other infections were detected during gestation. Despite the episode of fetal anoxia, the patient did not show any signs of retinopathy of prematurity (ROP) or any other hypoxia/ventilation-associated findings such as pathological neovascularization. There was no use of forceps during delivery and no coagulation disorders were detected. Dengue virus infection was confirmed by the identification of the NS1 protein (ELISA) in both mother and child.

Thus, based on the clinical presentation of mother and child, vertical transmission of dengue virus with intraocular involvement manifested by mild vitritis, cotton wool spots and vitreous and preretinal hemorrhages in both eyes was our working hypothesis. In an observational study in adults diagnosed with dengue and maculopathy, patients had diffuse retinal thickening in 44.6% of cases, foveolitis in 33.8% and cystoid macular edema in 21.6%^(9)^. The patient, however, had a normal macular OCT as well as central subfield thickness (CSFT) within the standard for his age^(10)^. If macular changes occurred in our case, they probably regressed 16 weeks after admittance, when OCT examination was feasible.

Retinal and vitreous hemorrhages as well as small vessel occlusions may be the presenting signs of vertical transmission of dengue from mother to child. We thus recommend screening for dengue retinopathy in the offspring of a mother with a positive history of having acquired this disease late in pregnancy.
